# Combined Catheter Ablation and Left Atrial Appendage Closure in Atrial Fibrillation Patients with and without Prior Stroke

**DOI:** 10.1155/2021/2138670

**Published:** 2021-12-08

**Authors:** Bin-Feng Mo, Rui Zhang, Jia-Li Yuan, Jian Sun, Peng-Pai Zhang, Wei Li, Mu Chen, Qun-Shan Wang, Yi-Gang Li

**Affiliations:** Department of Cardiology, Xinhua Hospital Affiliated to Shanghai Jiao Tong University School of Medicine, #1665 Kong Jiang Road, Shanghai 200092, China

## Abstract

**Background:**

Combined atrial fibrillation (AF) ablation and left atrial appendage closure (LAAC) has been practiced for management of both the symptoms and the high stroke risk of AF. Data of the combined procedure in selected patients with prior stroke are limited. The aim of this study is to compare the safety and efficacy of combined catheter ablation and LAAC between AF patients with and without prior stroke.

**Methods and Results:**

This retrospective study enrolled 296 patients who underwent combined procedures of AF ablation and LAAC. Patients were divided into two groups: 81 patients with prior stroke (Stroke group) and 215 patients without prior stroke (Control group). Combined procedures were successfully performed in all the patients. Patients in the Stroke group had higher CHA_2_DS_2_-VASc scores (4.9 ± 1.2 vs. 3.2 ± 1.0, *P* < 0.001) and higher HAS-BLED scores (3.5 ± 1.1 vs. 3.0 ± 1.0, *P* < 0.001) compared with those in the Control group. Procedure-related complications in the Stroke group included two pericardial effusions and two groin hematomas, which did not differ significantly fromthe Control group (4.9% vs. 4.2%, *P*=0.778). After a mean follow-up of 20 months, the AF-free rate of the Stroke group was comparable with that of the Control group (64.2% vs. 68.4%, *P*=0.495). The relative risk reductions in stroke and bleeding (observed rate compared to that predicted from the CHA_2_DS_2_-VASc and HAS-BLED scores) were 80% and 79%, respectively, in the Stroke group, and 62% and 62%, respectively, in the Control group.

**Conclusions:**

The combination of catheter ablation and LAAC is safe and efficient in selected AF patients with prior stroke. It was observed that patients with prior stroke may benefit more from risk reductions of stroke and bleeding following the combined procedure.

## 1. Introduction

Atrial fibrillation (AF) is the most common atrial arrhythmia. One of the most severe complications of AF is ischemic stroke, and stroke caused by AF is associated with a poor prognosis [1]. Catheter ablation is an effective treatment for patients with symptomatic drug-refractory AF, but its role in long-term stroke prevention has not been well established [2]. Left atrial appendage closure (LAAC) has been proven as a safe and effective alternative to long-term anticoagulation in patients with an increased risk of stroke and bleeding [3]. Recently, the combined procedure of ablation for AF and LAAC for stroke prevention has attracted increasing attention in selected patients with symptomatic AF and a high risk of stroke. Several studies have reported the safety and feasibility of performing the combined procedure [4–7].

Patients surviving an initial stroke are at a significantly increased risk of further strokes compared to the general population. A few studies have reported the outcomes of either catheter ablation [8–10] or LAAC [11, 12] in patients with prior stroke. However, evidence is scarce with respect to the combined therapy of AF ablation and LAAC in patients with prior stroke. Thus, the aim of this study was to investigate the safety and efficacy of combined catheter ablation and LAAC in AF patients with prior stroke.

## 2. Methods

### 2.1. Study Population

This single-center retrospective study enrolled consecutive patients with nonvalvular AF who underwent a combined procedure of catheter ablation and LAAC between April 2017 and February 2019. All participants were included based on the following criteria: age > 18 years; symptomatic nonvalvular AF refractory to antiarrhythmic drugs; and with CHA_2_DS_2_-VAS score ≥ 2 plus one of the following situations: (1) high bleeding risk (HAS-BLED score ≥ 3); (2) history of stroke or systemic embolic event under oral anticoagulation (OAC) treatment; (3) intolerance to chronic OAC; and (4) preference for LAAC device implantation as an alternative to long-term OAC [2, 13, 14]. The exclusion criteria included valvular heart disease, previous AF ablation, recent myocardial infarction, and stroke within three months.

A total of 296 patients were enrolled. The cohort was divided into two groups: patients with prior stroke (the Stroke group, *n* = 81) and patients without prior stroke (the Control group, *n* = 215). This retrospective study was approved by the Ethics Committee of Xinhua Hospital Affiliated to Shanghai Jiao Tong University School of Medicine and complies with the Declaration of Helsinki. Written informed consent was obtained from each patient.

### 2.2. Preprocedural Assessment

All the procedures were performed in a high-volume AF center (>1000 cases of AF intervention per year) and undertaken by experienced operators who had passed the learning curves of either catheter ablation or LAAC. Left atrial appendage thrombus exclusion and size measurement were conducted by transesophageal echocardiography (TEE) before procedures. A cardiac computed tomography (CT) scan and 3-dimensional reconstruction of the left atrium were performed preprocedurally in 93.6% (277/296) of patients to assist catheter ablation and LAAC.

### 2.3. Combined Procedure

The combined procedure was performed as described previously [7]. AF ablation was performed before LAAC implantation. Under conscious sedation, a decapolar catheter was positioned in the coronary sinus and two transseptal accesses were obtained through the right femoral vein. Mapping and ablation were performed either under the guidance of CARTO (Biosense Webster, Diamond Bar, CA, USA) or Ensite (St. Jude Medical, St. Paul, MN, USA) 3-dimensional electroanatomic mapping systems. For patients with paroxysmal AF, standard pulmonary vein isolation was performed, and for those with persistent AF, additional linear and/or complex fragmented atrial electrogram ablations were performed according to the physician's discretion. Sinus rhythm was restored by either ablation or electric cardioversion.

LAAC was performed after AF ablation. The LAAC procedure was performed as described previously [15, 16]. In brief, the LAAC procedure was performed under local anesthesia and fluoroscopy guidance, and TEE was introduced under deep sedation after device deployment to reconfirm the position of the device before release. A mean left atrial pressure of above 10 mmHg was obtained after transseptal puncture. A WATCHMAN device (Boston Scientific, Marlborough, MA, USA) with an appropriate size (21, 24, 27, 30, and 33 mm) was chosen, generally, 10–30% oversizing based on the ostial width of the LAA measured by angiography or cardiac CT. The device was then advanced into the delivery sheath and deployed by sheath retraction guided by fluoroscopy. A preliminary assessment was performed by angiography and tug test under fluoroscopy to check the device position and stability. TEE was then performed to reconfirm the position with minimal (<5 mm) to no residual peridevice leaks and a proper compression ratio under deep sedation. The device was released if it was verified by the assessment of “PASS” criteria.

### 2.4. Postprocedural Anticoagulation

Patients received OAC therapy for at least 3 months following the procedure, unless there were contraindications. Dual antiplatelet therapy was recommended for another 3 months, and then life-long aspirin was prescribed if follow-up TEE showed either complete closure of the LAA or limited residual peridevice flow (jet <5 mm in width).

### 2.5. Follow-Up

After discharge, office or transtelephonic visits were scheduled for the 3rd month, 6th month, and 12th month following the procedure and once every half a year thereafter. ECG or 24 h Holter monitoring was performed at each office visit for patients. Antiarrhythmic drug therapy was discontinued after 3 months if no clinical or documented AF recurrences were identified. TEE was performed to assess the device occlusion safety and efficiency at 45 days of follow-up time points. Adverse events were reported during the follow-up visit, based on the percutaneous LAA occlusion Munich Consensus Document [14], including mortality, thromboembolic events (stroke and systemic embolism), and bleeding events.

### 2.6. Statistical Analysis

Continuous variables are described as mean ± standard deviation (median (interquartile range) for nonnormal data) and are compared using the Student's *t*-test (Mann–Whitney *U* test if normality is not satisfied). Categorical variables are presented as percentages and are analyzed using the chi-square test or Fisher exact test where appropriate. The observed stroke and bleeding event rates during follow-up are calculated as the number of events per 100 patient-years and are compared with the predicted event rates based on the CHA2DS2-VASc and HAS-BLED scores using published literature [17, 18]. All analyses were performed using SPSS version 22.0 (IBM Software Inc., Armonk, NY). Two-sided *P* values of <0.05 were considered statistically significant.

## 3. Results

### 3.1. Baseline Characteristics

A total of 81 patients in the Stroke group and 215 patients in the Control group were included. Mean ages were 69.6 ± 8.2 years and 68.9 ± 7.9 years in the two groups (Table 1). Indications for LAAC are demonstrated in Table 1. Patients in the Stroke group had a higher risk for stroke based on the CHA_2_DS_2_-VAS score (4.9 ± 1.2 vs. 3.2 ± 1.0, *P* < 0.001) and had a higher bleeding risk based on the HAS-BLED score (3.5 ± 1.1 vs. 3.0 ± 1.0, *P* < 0.001) compared with those in the Control group. The other baseline characteristics were comparable between the two groups and are described in Table 1.

### 3.2. Procedural Characteristics

The periprocedural outcomes are given in Table 2. The procedure time (154.1 ± 24.9 min vs. 159.7 ± 28.6 min) and fluoroscopy time (10.2 ± 3.2 min vs. 10.9 ± 3.4 min) were comparable between the Stroke and Control groups. A total of 37 patients (45.7%) in the Stroke group and 106 patients (49.3%) in the Control group underwent standard pulmonary vein isolation only (*P*=0.578), while additional linear/CFAE ablations were performed in the rest of the patients. All patients in the two groups achieved a satisfactory seal (residual leak ≤5 mm). Complete occlusion was achieved in 95.1% of the Stroke group and 94.0% of the Control group (*P*=0.715).

There were 4 patients (4.9%) in the Stroke group with procedure-related complications. Two were pericardial effusions which required percutaneous drainage, and the other two were minor groin hematomas. In the control group, four (1.9%) had pericardial effusion and three (1.4%) had groin hematomas. One patient (0.5%) in the Control group suffered a transient coronary air embolism with chest pain and ST-segment elevation in inferior wall leads, which was resolved by forced coughing. Periprocedural stroke occurred in one patient in the Control group on the second day of the procedure and was confirmed by cranial CT. No significant difference was observed in the procedure-related complications between the two groups (4.9% vs. 4.2%, *P*=0.778).

### 3.3. Clinical Outcomes

Data on TEE imaging at least 45 days after the procedure were available in 77 patients (95.1%) of the Stroke group and 208 patients (96.7%) of the Control group. Eleven patients were evaluated by CT imaging. Satisfactory LAA occlusion (residual leak ≤5 mm) was noted in all patients in the Stroke group and 99.5% of patients in the Control group (*P*=0.539) (Table 3). One patient of the Control group had a residual leak >5 mm due to device migration and was continued on OAC. Device-related thrombosis was detected on TEE in one patient (1.2%) in the Stroke group and two patients (0.9%) in the Control group, which resolved without clinical sequelae on continued oral anticoagulation.

The average follow-up was 20.8 ± 7.0 months in the patients of the Stroke group and 20.2 ± 6.3 months in the patients of the Control group (Table 3). A total of 52 patients (64.2%) in the Stroke group and 147 patients (68.4%) in the Control group were AF-free (*P*=0.495) (Table 3). Fifteen patients (18.5%) in the Stroke group and 32 (14.9%) in the Control group who had an AF recurrence underwent a repeat ablation.

After the procedure, 93.8% of the Stroke group and 94.9% of the Control group were prescribed an OAC, while the rest of the patients were given antiplatelet therapy. During the latest follow-up, 3.7% of the Stroke group and 2.8% of the Control group remained on OAC, while antiplatelets were prescribed for 88.9% of the Stroke group (82.7% single and 6.2% dual) and 89.3% of the Control group (80.0% single and 9.3% dual) (Table 3). The remainder (7.4% and 7.9%) received no therapy.

**Figure 1:**
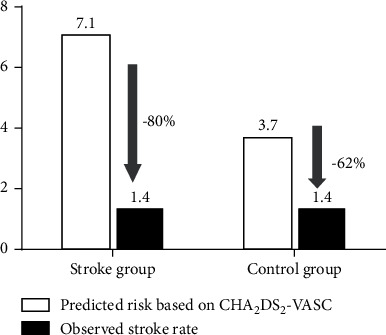
Efficacy in reduction of stroke rate (per 100 patients-year) during the overall follow-up.

**Figure 2:**
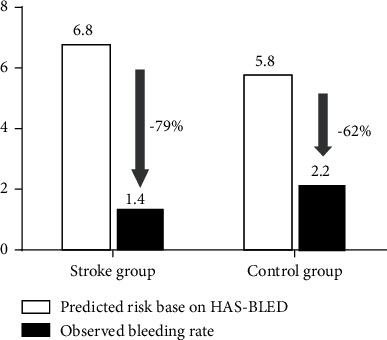
Efficacy in reduction of bleeding rate (per 100 patients-year) during the overall follow-up.

In the Stroke group, a total of two ischemic strokes and two gastrorrhagia were recorded at follow-up, resulting in an observed annualized stroke rate of 1.4% and an observed annualized bleeding rate of 1.4% (Table 3). In the Control group, five patients suffered ischemic stroke and eight had major bleeding events (gastrointestinal 4 (1.9%), pulmonary 1 (0.5%), urethral 1 (0.5%), and epistaxis 1 (0.5%)), resulting in an observed annualized stroke rate of 1.4% and an observed annualized bleeding rate of 2.2%. Compared with the expected stroke rate derived from the CHA_2_DS_2_-VASc score, an 80% annualized stroke reduction in the Stroke group and a 62% annualized stroke reduction in the Control group were observed (Figure 1). Annualized bleeding reduction of 79% in the Stroke group and 62% in the Control group was found compared to that expected from the HAS-BLED score (Figure 2).

## 4. Discussion

The current study presents a comparison between AF patients with and without prior stroke who underwent the combined procedures. The results demonstrate that combined therapy of catheter ablation and LAAC in selected AF patients with prior stroke is safe and efficient. Compared with patients without prior stroke, patients with prior stroke may benefit more from reduced risk of stroke and bleeding following the combined procedure.

AF is an independent risk factor for stroke, and stroke occurring with AF is more likely to be fatal or more severe than non-AF stroke [19]. OAC can significantly reduce stroke events and improve outcomes in AF patients with a high risk of stroke [20]. Catheter ablation for AF has been proven to be effective in rhythm control and improves the quality of life, but no randomized clinical trial has shown a reduction in long-term ischemic stroke [21]. On the contrary, LAAC with the WATCHMAN™ device has been demonstrated in randomized trials to reduce strokes and, therefore, can be an alternative to warfarin therapy for stroke prevention [22, 23]. The combined procedure of AF ablation and LAAC can provide concomitant rhythm control as well as stroke prevention in patients with symptomatic AF and a high risk of stroke [4–7].

The safety of combined ablation and LAAC was first reported by Swaans et al. in a small observational study of 30 patients in 2012 [4]. Pulmonary vein isolation and additional complex-fractionated atrial electrogram ablation were performed, followed by LAAC with the WATCHMAN device. Successful closure with satisfactory seals was achieved in all the patients with three (10%) patients experiencing minor periprocedural complications. During 1-year follow-up, 70% of the patients were free from atrial arrhythmias and no thromboembolic events occurred. A high procedural success rate with a relatively low complication rate of the combined procedure as well as satisfactory midterm follow-up results was obtained in that study.

Since then, a series of observational studies, including two multicenter registry studies with long-term follow-ups, further supported and strengthened the notion that the combined therapy can be feasible, safe, and successful [5, 6]. Wintgens et al. [5] published long-term follow-up results of a prospective real-world multicenter trial with a large patient cohort of 349 patients. Rates of satisfactory and complete LAA sealing were 100% and 92.6%, respectively. After 35 months of follow-up, 49% of the patients remained AF-free. Annualized stroke and major bleeding rates were 0.7% and 1.1%, respectively, referring a 78% risk reduction of stroke and a 71% risk reduction of bleeding. Phillips et al. [6] reported the long-term outcome results of 142 patients by pooling data from the EWOLUTION and WASP registries. Successful LAAC was achieved in 99.3% of the included patients, with a 97.2% complete LAA seal rate. The 30-day device and/or procedure-related SAE rate was 2.1%. After a mean follow-up of 726 ± 91 days, the annualized stroke and major bleeding rates were 1.09% and 1.09%, respectively. We recently reported the results of the combined procedure in a case-control study using the propensity score matching method [7]. Outcomes of the combined procedure with ablation alone and LAAC alone were compared. Procedure-related complications were similar among the groups. The AF-free rate was comparable between the ablation alone group and the combined procedure group (67.1% vs. 69.7%, *P* > 0.05). Complete occlusion rates were also similar between the LAAC alone group and the combined procedure group immediately postprocedure (94.7% vs. 93.4%) and at 45 days postprocedure (82.9% vs. 85.5%). The case-control results supplement the safety and efficacy of the combined therapy. Although the safety and efficacy of the combined procedure in ordinary patients with AF have been substantially demonstrated [4–7], evidence is scarce with respect to the feasibility of interventional therapies in AF patients with a previous stroke history. In this current study, we reported valuable evidence on the subgroup of patients with prior stroke and AF who underwent a combined procedure of ablation and LAAC.

Previous studies have reported the safety of patients with prior stroke undergoing AF catheter ablation [8, 9] or LAAC [11]. In the present study, we focused on selected patients with symptomatic AF and prior stroke who underwent combined therapy of catheter ablation and LAAC. We reported low periprocedural complications in patients with prior stroke which was comparable with those without prior stroke. Regarding AF recurrence, Li et al. reported a similar rate of AF recurrence between those with or without prior stroke during a near 2-year follow-up [8]. Our results demonstrated a comparable AF-free rate of 64.2% and 68.4% between patients with and without prior stroke following the combined procedure. These two consistent results suggest that a previous stroke seems not to have an impact on the success of AF ablation. Another study revealed that in patients with AF and a prior history of stroke, patients undergoing ablation have lower rates of recurrent stroke compared to AF patients not ablated with five years of follow-up [10], although the full mechanisms of benefit are not yet known.

Previous studies have shown that patients with prior cerebral embolic events are at an extremely high risk of recurrence of stroke [24, 25]. The cumulative risk of stroke was 11.1% at 1 year and up to 26.4% at 5 years for patients after initial stroke [26]. Published studies have described the effectiveness of LAAC in patients with AF and prior stroke as secondary prevention over short-term observation [12] and long-term observation [11]. In our study, patients with prior stroke had significantly higher CHA_2_DS_2_-VASc and HAS-BLED scores than those without, which meant a higher risk of stroke and bleeding. However, the observed annualized stroke rate and annualized bleeding rate were similar in patients with and without prior stroke in this study. Thus, a more significant risk reduction of stroke and bleeding compared to that expected from the risk scores in patients with prior stroke was observed, implying that patients with prior stroke are better candidates for the combined procedure of ablation and LAAC.

There are several limitations in our study. This is a single-center retrospective study with a moderate sample size. The history of stroke was self-reported which may underestimate the percentage of strokes in patients with AF. Follow-up with ECG and 24 h Holter recordings for detecting AF recurrence is another limitation. Asymptomatic arrhythmias or nondocumented symptomatic episodes may have been undetected.

## 5. Conclusions

In conclusion, combined therapy of AF ablation and LAAC is safe and efficient in patients with prior stroke. Long-term data and large-scale studies are needed to further verify the benefit of combined therapy in selected patients with AF and prior stroke.

## Figures and Tables

**Table 1:** Baseline characteristics of the study population.

	Stroke group *N* = 81	Control group *N* = 215	*P* value
Female	36 (44.4)	107 (49.8)	0.414
Age (years)	69.6 ± 8.2	68.9 ± 7.9	0.476
BMI (kg/m^2^)	24.5 ± 3.1	24.8 ± 3.4	0.492
Paroxysmal AF	40 (49.4)	97 (45.1)	0.512
Persistent AF	41 (50.6)	118 (54.9)	0.512
Coronary artery disease	15 (18.5)	49 (22.8)	0.426
Heart failure	17 (21.0)	58 (27.0)	0.291
Hypertension	64 (79.0)	175 (81.4)	0.643
Diabetes mellitus	18 (22.2)	65 (30.2)	0.171
Previous stroke	81 (100.0)	0 (0.0)	<0.001
Left atrial diameter (mm)	43.1 ± 5.1	42.5 ± 5.7	0.353
LVEF (%)	62.8 ± 6.5	63.6 ± 6.3	0.359
CHA_2_DS_2_-VASc score	4.9 ± 1.2	3.2 ± 1.0	<0.001
HAS-BLED score	3.5 ± 1.1	3.0 ± 1.0	<0.001
Indications for LAAC			<0.001
High bleeding risk	40 (49.4)	124 (57.7)	
History of stroke under OAC	21 (25.9)	0 (0.0)	
Intolerance to chronic OAC	7 (8.6)	22 (10.2)	
Patient preference	13 (16.0)	69 (32.1)	

Values are mean ± SD or *n* (%) as appropriate. AF, atrial fibrillation; BMI, body mass index; LAAC, left atrial appendage closure; LVEF, left ventricular ejection fraction; OAC, oral anticoagulation.

**Table 2:** Procedural characteristics and safety.

	Stroke group *N* = 81	Control group *N* = 215	*P* value
Procedure time (min)	154.1 ± 24.9	159.7 ± 28.6	0.123
Fluoroscopy time (min)	10.2 ± 3.2	10.9 ± 3.4	0.117
PVI only	37 (45.7)	106 (49.3)	0.578
PVI plus linear/CFAE ablation	44 (54.3)	109 (50.7)	0.578
Morphology of LAA			
Cauliflower	48 (59.3)	133 (61.9)	0.682
Chicken wing	18 (22.2)	40 (18.6)	0.485
Cactus	8 (9.9)	27 (12.6)	0.524
Windsock	7 (8.6)	15 (7.0)	0.626
LAA ostium width (mm)	22.5 ± 3.0	23.1 ± 3.4	0.165
Device size (mm)	28.1 ± 3.1	28.6 ± 3.4	0.253
Device compression (%)	20.0 ± 4.8	19.3 ± 5.4	0.292
Successful implantation	81 (100)	215 (100)	—
Peridevice leak at implantation			
Complete occlusion of LAA	77 (95.1)	202 (94.0)	0.715
Leak ≤ 5 mm	4 (4.9)	13 (6.0)	0.715
Leak > 5 mm	0 (0.0)	0 (0.0)	—
Procedure-related complications	4 (4.9)	7 (4.2)	0.778
Death	0 (0.0)	0 (0.0)	—
Pericardial effusion	2 (2.5)	4 (1.9)	0.740
Coronary air embolism	0 (0.0)	1 (0.5)	0.539
Stroke	0 (0.0)	1 (0.5)	0.539
Major bleeding events	0 (0.0)	0 (0.0)	—
Complications of vascular access	2 (2.5)	3 (1.4)	0.523

Values are mean ± SD or *n* (%) as appropriate. AF, atrial fibrillation; CFAE, complex fragmented atrial electrogram; LAA, left atrial appendage; PVI, pulmonary vein isolation.

**Table 3:** Outcomes at follow-up.

	Stroke group *N* = 81	Control group *N* = 215	*P* value
Average follow-up (months)	20.8 ± 7.0	20.2 ± 6.3	0.507
Peridevice leak at 45 days follow-up			
Complete occlusion of LAA	66 (81.5)	165 (76.7)	0.380
Leak ≤ 5 mm	15 (18.5)	49 (22.8)	0.426
Leak > 5 mm	0 (0.0)	1 (0.5)	0.539
Device-associated thrombosis	1 (1.2)	2 (0.9)	0.816
AF-free at follow-up			
Overall	52 (64.2)	147 (68.4)	0.495
Paroxysmal AF	28 (70.0)	73 (75.3)	0.525
Persistent AF	24 (58.5)	74 (62.7)	0.636
Redo ablation	15 (18.5)	32 (14.9)	0.446
Antithrombotic medications at latest follow-up			
Oral anticoagulation	3 (3.7)	6 (2.8)	0.683
Dual antiplatelet therapy	5 (6.2)	20 (9.3)	0.388
Single antiplatelet therapy	67 (82.7)	172 (80.0)	0.597
None	6 (7.4)	17 (7.9)	0.866
Thromboembolic events	2 (2.5)	5 (2.3)	0.942
Ischemic stroke	2 (2.5)	5 (2.3)	0.942
Systemic embolism	0 (0.0)	0 (0.0)	—
Observed annualized stroke rate (%)	1.4	1.4	—
Major bleeding	2 (2.5)	8 (3.7)	0.595
Intracranial	0 (0.0)	0 (0.0)	—
Gastrointestinal	2 (2.5)	4 (1.9)	0.740
Pericardial	0 (0.0)	0 (0.0)	—
Pulmonary	0 (0.0)	1 (0.5)	0.539
Urethral	0 (0.0)	2 (0.9)	0.385
Epistaxis	0 (0.0)	1 (0.5)	0.539
Observed annualized bleeding rate (%)	1.4	2.2	—
Mortality	1 (1.2)	2 (0.9)	0.816
Cardiovascular	1 (1.2)	1 (0.5)	0.472
Noncardiovascular	0 (0.0)	0 (0.0)	—
Reason unknown	0 (0.0)	1 (0.5)	0.539

Values are mean ± SD or *n* (%) as appropriate. AF, atrial fibrillation; LAA, left atrial appendage; PVI, pulmonary vein isolation; TEE, transesophageal echocardiography.

## Data Availability

The data used to support the findings of this study are available from the corresponding author upon request.

## Abbreviations

AF: Atrial fibrillation
CT: Computed tomography
LAAC: Left atrial appendage closure
OAC: Oral anticoagulation
TEE: Transesophageal echocardiography.
